# Suicide Risk Among Veterans Who Receive Evidence-Based Therapy for Posttraumatic Stress Disorder

**DOI:** 10.1001/jamanetworkopen.2024.52144

**Published:** 2024-12-26

**Authors:** Kevin G. Saulnier, Stuart Brabbs, Benjamin R. Szymanski, Ilan Harpaz-Rotem, John F. McCarthy, Rebecca K. Sripada

**Affiliations:** 1 Serious Mental Illness Treatment Resource and Evaluation Center, Office of Mental Health, Department of Veterans Affairs, Ann Arbor, Michigan; 2 Department of Psychiatry, University of Michigan, Ann Arbor; 3 Center for Clinical Management Research, Health Services Research & Development, Department of Veterans Affairs, Ann Arbor, Michigan; 4 Veterans Affairs Northeast Program Evaluation Center, West Haven, Connecticut; 5 Veterans Affairs National Center for PTSD, Veteran Affairs Connecticut Healthcare System, West Haven, Connecticut; 6 Department of Psychiatry, Yale University, New Haven, Connecticut; 7 Office of Suicide Prevention, Department of Veterans Affairs, Washington, DC

## Abstract

**Importance:**

Suicide rates are increased for veterans with posttraumatic stress disorder (PTSD). To inform Veterans Health Administration (VHA) operations, it is important to assess whether suicide risk for veterans differs by receipt of evidence-based treatments.

**Objective:**

To assess suicide risks among veterans in VHA care with new PTSD diagnoses who initiated or received an adequate course of evidence-based psychotherapy (cognitive processing therapy or prolonged exposure [CPT/PE]).

**Design, Setting, and Participants:**

This cohort study of veterans in VHA care evaluated suicide risk through 2020 for veterans with initial PTSD diagnoses during 2016-2019. Data collection and analyses were performed from March 22 to November 22, 2023. The population consisted of veterans who received an initial PTSD diagnosis (preceded by at least 12 months without a PTSD diagnosis) in 2016-2019.

**Exposures:**

Following the index PTSD diagnosis, receipt of a CPT/PE encounter and receipt of an adequate course of therapy (≥8 CPT/PE encounters).

**Main Outcome and Measure:**

Suicide mortality, per death certificate data from the Veterans Affairs/Department of Defense Mortality Data Repository.

**Results:**

Of the population-based sample of 847 217 US veterans, most were male (735 974 [86.9%]); the mean (SD) age was 50.1 (16.3) years. From initial PTSD diagnosis through 2020, there were 1552 suicides. Multivariable proportional hazards regression indicated that initiation of CPT/PE treatment was associated with a 23% lower suicide risk compared with those who did not initiate CPT/PE (hazard ratio [HR], 0.77; 95% CI, 0.59-0.99). Receipt of an adequate course was not associated with suicide (HR, 0.80; 95% CI, 0.55-1.18). Similar results were observed when limiting the cohort to those who received any psychotherapy (n = 552 742; CPT/PE initiation: HR, 0.73; 95% CI, 0.56-0.95; adequate course: HR, 0.77; 95% CI, 0.52-1.12).

**Conclusions and Relevance:**

The findings of this study suggest that, among veterans in VHA care with new PTSD diagnoses, CPT/PE initiation was associated with lower suicide risk. However, for those who initiated CPT/PE, receipt of 8 or more sessions was not associated with added benefit.

## Key Points

**Question:**

Are initiation and an adequate course of evidence-based therapy (cognitive processing therapy or prolonged exposure [CPT/PE]) sessions for posttraumatic stress disorder (PTSD) associated with suicide risk among veterans with new PTSD diagnoses?

**Findings:**

In a cohort study of 847 217 veterans with initial Veterans Health Administration PTSD diagnoses in 2016-2019, proportional hazards regressions that adjusted for propensity to initiate CPT/PE therapy and veteran characteristics indicated that CPT/PE initiation was associated with lower suicide risk through 2020. An adequate course of therapy was not associated with suicide risk.

**Meaning:**

The findings of this study suggest that veterans who initiated CPT/PE had a lower suicide risk than those who did not.

## Teaser

This cohort study examines the association of initiation and an adequate course of cognitive processing therapy or prolonged exposure with suicide risk among veterans with posttraumatic stress disorder.

## Introduction

Suicide prevention is the top clinical priority of the Department of Veterans Affairs (VA),^1^ the largest integrated health system in the US. As part of measurement-based management, the VA evaluates the impact of suicide prevention initiatives and clinical practices.^2^ Posttraumatic stress disorder (PTSD) is more prevalent among veterans (lifetime prevalence, 9.4%)^3^ than nonveteran adults (lifetime prevalence, 6.1%).^4^ Posttraumatic stress disorder is associated with increased rates of veteran suicide.^5,6,7,8^ The PTSD Clinical Practice Guidelines were developed by the VA and Department of Defense based on findings that specific treatments reduced PTSD symptoms.^9^ However, to date we are not aware of studies evaluating whether recommended PTSD treatment receipt is associated with suicide in the VHA. As part of ongoing VA suicide prevention efforts,^10^ it is important to assess whether suicide risks differ in association with initiation of and with an adequate amount of PTSD treatment for veterans receiving VHA care.

Recommended first-line treatments for PTSD include cognitive processing therapy (CPT)^11^ and prolonged exposure (PE).^9,12^ Given strong support for CPT and PE, as reflected by meta-analytic evidence that supports CPT/PE compared with other PTSD psychotherapies,^13,14^ the VHA implemented CPT and PE nationally in 2007.^15^ Each treatment has been found to reduce veteran distress and disability,^16,17,18,19,20,21^ including among veterans with PTSD and current suicidal ideation.^22^ Studies of veteran and active duty military personnel, with relatively small samples (20-335 individuals), suggest that receipt of CPT and PE has been associated with decreased suicidal ideation.^23,24,25,26,27,28^ However, despite evidence documenting the efficacy and effectiveness of CPT/PE in reducing PTSD symptoms, to our knowledge, no large-scale study has examined how initiation of these treatments or receipt of an adequate amount of therapy may be associated with veteran suicide risk.

To support VHA clinically based suicide prevention activities and enhance clinical operations, this study evaluated suicide risks in association with evidence-based treatment initiation and longer-term treatment engagement among veterans in VHA care with initial PTSD diagnoses. We assessed possible associations between suicide and initiation of CPT/PE treatment and receipt of at least 8 sessions of CPT/PE treatment, which is considered an adequate course.^29^ We hypothesized that initiation of CPT/PE and receipt of an adequate course of therapy were negatively associated with suicide risk. Furthermore, given findings from meta-analyses of prior studies,^13,14^ we hypothesized that, specifically among veterans who received some VHA psychotherapy, suicide risks were lower among those who received CPT/PE.

## Methods

This study was conducted as part of ongoing operations and quality improvement work in the VA Office of Suicide Prevention and the Office of Mental Health. Therefore, this work was exempt from institutional review board approval as operations evaluations for quality improvement are conducted under a waiver of consent. In preparing this article, we followed the Strengthening the Reporting of Observational Studies in Epidemiology (STROBE) reporting guideline for cohort studies.^30^

### Data Sources

Demographic, clinical, and treatment receipt data were drawn from the VHA Corporate Data Warehouse. Vital status and cause of death data were received from the VA and Department of Defense Mortality Data Repository.

### Cohort

Patients who were veterans receiving treatment through the VHA were included if they had a new *International Statistical Classification of Diseases and Related Health Problems, Tenth Revision* (*ICD-10*)^31^ diagnosis of PTSD (codes F43.10, F43.11, and F43.12) documented in VHA records from January 1, 2016, through December 31, 2019. New PTSD diagnoses were defined as a PTSD diagnosis without a VHA PTSD diagnosis documented in the previous 365 days. Veteran status was identified based on VA and Department of Defense administrative records requiring activation for federal military service. Veterans were assigned an index date corresponding to the date of their first new PTSD diagnosis. Analyses excluded veterans with a documented date of death before the index date, documented location of death in Puerto Rico or the Virgin Islands, missing or invalid data on patient age (ie, ages <18 or >115 years), no recent VHA use in the previous year and never submitted to the Mortality Data Repository, and missing covariates of interest. Of veterans meeting these criteria, 847 217 (96.7%) had complete data and were included in the analyses.

### Measures

#### Receipt of Evidence-Based Treatment for PTSD

Corporate Data Warehouse data were used to assess CPT/PE receipt throughout the follow-up period. Receipt of CPT/PE was determined by Current Procedural Terminology codes for psychotherapy (90834, 90836, 90837, 90838, and 90853) accompanied by a medical record indicator that is populated when health care professionals use the national CPT/PE note templates.^32^ Initiation of CPT/PE was defined as the receipt of any CPT or PE sessions. Adequate CPT/PE therapy was defined as the receipt of 8 or more sessions of CPT or PE within a 180-day period because these treatments are designed to be delivered in a time-limited fashion (eg, 8-12 sessions), with a full course of CPT/PE generally requiring 8 to 9 sessions^11,12,33^ and many patients reporting clinically significant improvements within 8 sessions.^34,35,36^ Recognizing this time course, many studies of psychotherapy adequacy use 8 sessions as a metric for minimally adequate therapy,^37,38,39^ and 8 sessions is used as a mental health performance measure in VHA.

#### Suicide Mortality

Suicide mortality was measured from the index day until death or December 31, 2020, whichever came first. Suicide death was identified by *ICD-10* cause of death codes U03, X60 to X84, and Y87.0.

#### Covariates

Covariates were selected based on earlier work^6,8,40^ and theoretically and empirically derived measures that might influence the likelihood to receive CPT/PE.^41,42,43^ Covariates extracted from the Corporate Data Warehouse included demographic characteristics (age, sex, marital status, race and ethnicity, rurality, service connection, distance from home address to the nearest VHA facility, and period of military service documented in the VHA electronic medical records), contextual factors (encounter setting on the index diagnosis date, the VHA administrative parent facility where the index PTSD diagnosis was recorded, and the proportion of veterans at their VHA administrative parent facility with a PTSD diagnosis who received a PTSD Checklist for *Diagnostic and Statistical Manual of Mental Disorders, Fifth Edition* [PCL-5]^44^ screening), documentation of having experienced military sexual trauma, and physical health comorbidities (calculated using the Charlson comorbidity index^45^). The categories for race were Asian, Black, Native American, Native Hawaiian and Pacific Islander, White, and unknown race. The categories for ethnicity were Hispanic, non-Hispanic, and unknown ethnicity. Race and ethnicity were included as covariates in the multivariable proportional hazards regression models to control for known differences in suicide risk across these patient populations.

Mental health treatment covariates in the 12 months before the index date included the presence of inpatient mental health stays, number of inpatient mental health bed days, presence of emergency department visits, and psychotropic medication receipt. Mental health comorbidities included psychiatric conditions (substance use disorders, anxiety disorders, borderline personality disorder, depression, schizophrenia, and other mental health conditions), indication of a suicide attempt, Patient Health Questionnaire-9^46^ scores, positive Patient Health Questionnaire-2^47^ screens, and PCL-5 scores in the 12 months before the index date. To adjust for suicide risk, scores from the suicide predictive model^40^ incorporated in the VHA Recovery Engagement and Coordination for Health–Veterans Enhanced Treatment^48^ program for the month of the index PTSD diagnosis were included.

### Statistical Analysis

Analyses were conducted from March 22 to November 22, 2023, using SAS, version 8.3 (SAS Institute Inc).^49^ Frequencies were generated to characterize the demographic characteristics of the overall sample (N = 847 217) and by number of CPT/PE sessions attended. Next, proportional hazards regression models were estimated after adjusting for the propensity to receive CPT/PE. Propensity scores were used to address possible selection bias resulting from nonrandom treatment initiation across VHA subpopulations that may be associated with unmeasured PTSD severity or suicide risk. Propensity to receive CPT/PE was calculated with a logistic regression model that used inverse probability of treatment weighting (eTable 1, eTable 2, and eFigure in Supplement 1).^50,51,52^ The covariate distribution after inverse propensity weighting was balanced for all covariates except age, Charlson comorbidity scores, Primary Care PTSD Screen for *DSM-5* screen scores, and service era. The logistic regression model used to generate the propensity scores included all of the covariates described herein. By weighting using propensity scores in the proportional hazards regression models, we controlled for potential confounding due to variability in the likelihood of receiving CPT/PE.

In proportional hazards regression analyses, risk time began on the index diagnosis date and continued until death or December 31, 2020, whichever occurred first. Partially conditional proportional hazards regression models were used to control for nonsuicide mortality as a competing risk. Covariance sandwich estimators were used to adjust for the nested nature of the data, with patients clustered within facilities. Eight proportional hazards regression models were used to examine the associations between suicide and CPT/PE initiation and adequate course of therapy. Across models of CPT/PE initiation, CPT/PE initiation was entered as a time-varying binary indicator (0 before CPT/PE initiation or for veterans without initiation and 1 from the day of the first CPT/PE encounter and thereafter). For models of adequate CPT/PE course, adequate course was entered as a time-varying binary indicator (0 before the eighth CPT/PE session and 1 on the day of the eighth CPT/PE session and thereafter). First, univariate models were estimated in the overall cohort to examine the possible associations between suicide and CPT/PE initiation and an adequate course of therapy. Then, multivariable models were estimated in the overall cohort to examine possible associations between suicide risk and CPT/PE initiation and an adequate course of therapy, controlling for the number of mental health inpatient days in the prior 12 months and veteran age, sex, race and ethnicity, geographic region, rurality, and marital status. The cohort was next limited to veterans who received any type of psychotherapy during the follow-up period. Univariate models were estimated in this subset of the cohort to examine the possible associations between suicide and CPT/PE initiation and an adequate course of therapy. Multivariable models were then estimated in the subset of the cohort that received psychotherapy to examine the possible associations between suicide and CPT/PE initiation and CPT/PE an adequate course of therapy after controlling for the number of mental health inpatient days in the prior year and demographic variables. In models 5 to 8, risk time started at the initial psychotherapy session rather than the index date to more directly compare CPT/PE with other psychotherapies. Statistical significance was set at 2-tailed *P* < .05.

## Results

### Descriptive Statistics

Of the 847 217 veterans included in this analysis, 735 974 (86.9%) were male, 111 243 (13.1%) were female, 12 848 (1.5%) were Asian, 200 912 (23.7%) were Black, 14 813 (1.8%) were Native American, 12 025 (1.4%) were Native Hawaiian and Pacific Islander, 579 830 (68.4%) were White, and 26 789 (3.2%) were of unknown race, 323 664 (38.2%) were between ages 55 and 74 years (mean [SD], 50.1 [16.3] years), and 394 982 (46.9%) resided in the South (Table 1). Most patients received some psychotherapy (552 742 [65.2%]). A total of 73 473 individuals (8.7%) initiated CPT/PE, with 33 931 of those patients (46.2% of those with any CPT/PE) attending 8 or more sessions and being classified as having received an adequate course of therapy. Of the cohort, 1552 individuals (0.2%) died by suicide during the follow-up period.

**Table 1.** Characteristics of Veterans With New VHA PTSD Diagnoses, 2016-2019

Variable	No. (%)				
	Full cohort (N = 847 217)	0 CPT/PE sessions (n = 773 744)	1-3 CPT/PE sessions (n = 10 129)	4-7 CPT/PE sessions (n = 29 413)	≥8 CPT/PE sessions (n = 33 931)
Age range, y					
18-34	193 412 (22.8)	171 753 (22.2)	3474 (34.3)	9493 (32.3)	8692 (25.6)
35-54	295 082 (34.8)	261 953 (33.9)	4614 (45.6)	13 212 (44.9)	15 303 (45.1)
55-74	323 664 (38.2)	305 519 (39.5)	1971 (19.5)	6576 (21.3)	9675 (28.5)
75-115	35 059 (4.1)	34 519 (4.5)	70 (0.7)	209 (0.7)	261 (0.8)
Sex					
Male	735 974 (86.9)	678 298 (87.7)	8119 (80.2)	22 935 (78.0)	26 622 (78.5)
Female	111 243 (13.1)	95 446 (12.3)	2010 (19.8)	6478 (22.0)	7309 (21.5)
Race^a^					
Asian	12 848 (1.5)	11 672 (1.5)	175 (1.7)	433 (1.5)	568 (1.7)
Black	200 912 (23.7)	181 699 (23.5)	2773 (27.4)	7761 (26.7)	8579 (25.3)
Native American	14 813 (1.8)	13 507 (1.8)	183 (1.8)	520 (1.8)	603 (1.8)
Native Hawaiian and Pacific Islander	12 025 (1.4)	11 032 (1.4)	158 (1.6)	425 (1.4)	410 (1.2)
White	579 830 (68.4)	531 624 (68.7)	6448 (63.7)	19 106 (65.0)	22 652 (66.8)
Unknown	26 789 (3.2)	24 210 (3.1)	392 (3.9)	1068 (3.6)	1119 (3.3)
Ethnicity^a^					
Hispanic	81 672 (9.6)	73 675 (9.5)	1210 (12.0)	3344 (11.4)	3443 (10.2)
Non-Hispanic	748 298 (88.3)	684 080 (88.4)	8733 (86.2)	25 579 (87.0)	29 906 (88.1)
Unknown	17 247 (2.0)	15 989 (92.7)	186 (1.8)	490 (1.6)	582 (1.7)
Region					
Northeast	97 649 (11.6)	91 058 (11.8)	872 (8.7)	2594 (8.9)	3125 (9.3)
Midwest	138 822 (16.5)	123 082 (16.0)	1998 (19.9)	6418 (21.9)	7324 (21.8)
South	394 982 (46.9)	361 129 (46.9)	4964 (49.3)	13 881 (47.5)	15 008 (44.6)
West	211 556 (25.1)	194 775 (25.3)	2231 (22.2)	6361 (21.7)	8189 (24.3)
Rurality					
Urban	599 731 (70.8)	545 497 (70.5)	7536 (74.4)	21 547 (73.3)	25 151 (74.1)
Rural	221 636 (26.2)	204 109 (26.4)	2371 (23.4)	7186 (24.4)	7970 (23.5)
Highly rural or insular island	25 850 (3.0)	24 138 (3.1)	222 (2.2)	680 (2.3)	810 (2.4)
Marital Status					
Married	442 360 (52.2)	404 678 (52.3)	5144 (50.8)	14 806 (50.3)	17 732 (52.3)
Never married	155 141 (18.3)	139 817 (18.1)	2129 (21.0)	6261 (21.3)	6934 (20.4)
Divorced	193 101 (22.8)	176 855 (22.9)	2235 (22.1)	6595 (22.4)	7416 (21.9)
Separated	39 294 (4.6)	35 815 (4.6)	517 (5.1)	1466 (5.0)	1496 (4.4)
Widowed	17 321 (2.0)	16 579 (2.1)	104 (1.0)	285 (1.0)	353 (1.0)
Suicide death	1552 (0.2)	1474 (0.2)	NA^b^	35 (0.1)	34 (0.1)

Abbreviations: CPT, cognitive processing therapy; NA, not applicable; PE, prolonged exposure; PTSD, posttraumatic stress disorder; VHA, Veterans Health Administration.

^a^ Data on race and ethnicity were extracted from the US Veterans Affairs Corporate Data Warehouse. No further breakdown of the classifications is available.

^b^ Fewer than 10 patients who attended 1 to 3 CPT/PE sessions died by suicide. Therefore, the sample and percentage values for this subgroup were suppressed per privacy guidelines.

### Proportional Hazards Regression Models Among the Full Cohort

Analysis of bivariate models for CPT/PE initiation and adequate therapy was conducted for all 847 217 study participants. Initiation of CPT/PE (HR, 0.79; 95% CI, 0.73-0.85; *P* < .001) and an adequate course of treatment (HR, 0.80; 95% CI, 0.72-0.89; *P* < .001) were associated with decreased suicide risk after controlling for the propensity to receive CPT/PE.

Multivariable models are presented in Table 2 for CPT/PE initiation and adequate course of therapy. After controlling for the propensity to receive CPT/PE, the number of inpatient mental health days in the year before the index date, and demographic variables, CPT/PE initiation (HR, 0.77; 95% CI, 0.59-0.997) was associated with a 23% lower suicide risk. In the model of CPT/PE initiation, increased suicide risk was associated with number of psychiatric inpatient days (HR, 1.01; 95% CI, 1.00-1.01); residence in the Midwest (HR, 1.37; 95% CI, 1.02-1.84), South (HR, 1.41; 95% CI, 1.10-1.81), and West (HR, 1.50; 95% CI, 1.17-1.9) vs the Northeast; and never married (HR, 1.37; 95% CI, 1.15-1.64) and divorced (HR, 1.55; 95% CI, 1.31-1.84) marital status. Decreased suicide risk was associated with being aged 35 to 54 years (HR, 0.76; 95% CI, 0.65-0.88) or 55 to 74 years (HR, 0.50; 95% CI, 0.43-0.59 vs age 18 to 34 years; female sex (HR, 0.44; 95% CI, 0.34-0.57); Asian (HR, 0.42; 95% CI, 0.25-0.71), Black (HR, 0.30; 95% CI, 0.23-0.38), and unknown (HR, 0.65; 95% CI, 0.44-0.95) race vs White race; and Hispanic ethnicity (HR, 0.54; 95% CI, 0.42-0.68). Adequate CPT/PE therapy was not associated with suicide risk after controlling for covariates (HR, 0.80; 95% CI, 0.55-1.18).

**Table 2.** Multivariable Proportional Hazards Regression Model of Suicide Mortality by Receipt of CPT/PE, Weighted by the Inverse Propensity to Receive CPT/PE Among 843 009 Patients

Variable	CPT/PE initiation		CPT/PE adequate therapy	
	HR (95% CI)	*P* value	HR (95% CI)	*P* value
CPT/PE receipt	0.77 (0.59-.997)	.05	0.80 (0.55-1.18)	.26
MH inpatient days	1.01 (1.00-1.01)	<.001	1.01 (1.00-1.01)	<.001
Age range, y				
18-34	1 [Reference]	NA	1 [Reference]	NA
35-54	0.76 (0.65-0.88)	<.001	0.76 (0.66-0.85)	<.001
55-74	0.50 (0.43-0.59)	<.001	0.50 (0.43-0.59)	<.001
≥75	0.91 (0.65-1.27)	.57	0.91 (0.65-1.28)	.60
Sex				
Male	1 [Reference]	NA	1 [Reference]	NA
Female	0.44 (0.34-0.57)	<.001	0.44 (0.34-0.57)	<.001
Race^a^				
Asian	0.42 (0.25-0.71)	.001	0.42 (0.25-0.71)	.001
Black	0.30 (0.23-0.38)	<.001	0.30 (0.23-0.38)	<.001
Native American	0.57 (0.32-1.02)	.06	0.57 (0.32-1.02)	.06
Native Hawaiian and Pacific Islander	0.64 (0.34-1.20)	.16	0.64 (0.34-1.20)	.16
White	1 [Reference]	NA	1 [Reference]	NA
Unknown	0.65 (0.44-0.95)	.03	0.65 (0.44-0.95)	.03
Ethnicity^a^				
Hispanic	0.54 (0.42-0.68)	<.001	0.54 (0.42-0.68)	<.001
Non-Hispanic	1 [Reference]	NA	1 [Reference]	NA
Unknown	1.09 (0.67-1.75)	.74	1.09 (0.67-1.75)	.73
Region				
Northeast	1 [Reference]	NA	1 [Reference]	NA
Midwest	1.37 (1.02-1.84)	.03	1.36 (1.02-1.83)	.04
South	1.41 (1.10-1.81)	.007	1.41 (1.10-1.81)	.007
West	1.50 (1.17-1.9)	.002	1.50 (1.16-1.94)	.002
Rurality				
Urban	1 [Reference]	NA	1 [Reference]	NA
Rural	0.97 (0.83-1.12)	.65	0.97 (0.83-1.12)	.97
Highly rural or insular island	0.85 (0.58-1.25)	.41	0.85 (0.58-1.25)	.41
Marital status				
Married	1 [Reference]	NA	1 [Reference]	NA
Never married	1.37 (1.15-1.64)	.001	1.37 (1.15-1.64)	<.001
Divorced	1.55 (1.31-1.84)	<.001	1.55 (1.31-1.84)	<.001
Separated	1.24 (0.93-1.69)	.16	1.25 (0.92-1.64)	.156
Widowed	1.16 (0.74-1.84)	.52	1.16 (0.74-1.84)	.52

Abbreviations: CPT, cognitive processing therapy; HR, hazard ratio; MH, mental health; NA, not applicable; PE, prolonged exposure.

^a^ Data on race and ethnicity were extracted from the US Veterans Affairs Corporate Data Warehouse. No further breakdown of the classifications is available.

### Proportional Hazards Regression Models Among Veterans Who Received Psychotherapy

Bivariate models were used to conduct analysis of 552 742 veterans who received any psychotherapy for CPT/PE initiation and an adequate course of therapy. Initiation of CPT/PE (HR, 0.68; 95% CI, 0.63-0.73; *P* < .001) and adequate course of therapy (HR, 0.78; 95% CI, 0.70-0.87; *P* < .001) were associated with decreased suicide risk after controlling for the propensity to receive CPT/PE.

Multivariable models are presented in Table 3 for CPT/PE initiation and an adequate course of therapy. After controlling for the propensity to receive CPT/PE and other covariates, CPT/PE initiation (HR, 0.73; 95% CI, 0.56-0.95) was associated with decreased suicide risk. Adequate therapy was not associated with suicide risk after controlling for covariates (HR, 0.77; 95% CI, 0.52-1.12).

**Table 3.** Multivariable Proportional Hazards Regression Model of Suicide Mortality by Receipt of CPT/PE, Weighted by the Inverse Propensity to Receive CPT/PE Among 549 541 Patients Who Received Psychotherapy

Variable	CPT/PE initiation		CPT/PE adequate therapy	
	HR (95% CI)	*P* value	HR (95% CI)	*P* value
CPT/PE receipt	0.73 (0.56-0.95)	.02	0.77 (0.52-1.12)	.17
MH inpatient days	1.01 (1.00-1.01)	<.001	1.01 (1.00-1.01)	<.001
Age, y				
18-34	1 [Reference]	NA	1 [Reference]	NA
35-54	0.75 (0.63-0.89)	.001	0.75 (0.63-0.89)	.001
55-74	0.44 (0.36-0.54)	<.001	0.44 (0.36-0.55)	<.001
≥75	0.46 (0.26-0.83)	.009	0.46 (0.26-0.84)	.01
Sex				
Male	1 [Reference]	NA	1 [Reference]	NA
Female	0.44 (0.30-0.54)	<.001	0.40 (0.30-0.53)	<.001
Race^a^				
Asian	0.40 (0.20-0.81)	.01	0.40 (0.20-0.81)	.01
Black	0.31 (0.24-0.40)	<.001	0.31 (0.24-0.40)	<.001
Native American	0.78 (0.40-1.54)	.47	0.78 (0.40-1.54)	.47
Native Hawaiian and Pacific Islander	0.27 (0.12-0.59)	.001	0.27 (0.12-0.59)	.001
White	1 [Reference]	NA	1 [Reference]	NA
Unknown	0.59 (0.36-0.96)	.03	0.59 (0.36-0.96)	.03
Ethnicity^a^				
Hispanic	0.60 (0.45-0.80)	.001	0.60 (0.45-0.80)	<.001
Non-Hispanic	1 [Reference]	NA	1 [Reference]	NA
Unknown	1.04 (0.56-1.91)	.91	1.04 (0.56-1.92)	.90
Region				
Northeast	1 [Reference]	NA	1 [Reference]	NA
Midwest	1.22 (0.87-1.71)	.26	1.21 (0.86-1.69)	.28
South	1.34 (0.99-1.81)	.06	1.34 (0.99-1.80)	.06
West	1.44 (1.05-1.96)	.02	1.43 (1.05-1.96)	.02
Rurality				
Urban	1 [Reference]	NA	1 [Reference]	NA
Rural	0.99 (0.83-1.19)	.91	0.99 (0.83-1.19)	.92
Highly rural or insular island	0.94 (0.58-1.51)	.79	0.94 (0.58-1.52)	.79
Marital status				
Married	1 [Reference]	NA	1 [Reference]	NA
Never married	1.36 (1.11-1.67)	.003	1.37 (1.11-1.68)	.003
Divorced	1.29 (1.06-1.57)	.01	1.29 (1.06-1.57)	.01
Separated	1.30 (0.90-1.87)	.17	1.30 (0.90-1.88)	.16
Widowed	1.50 (0.87-2.59)	.14	1.50 (0.87-2.60)	.14

Abbreviations: CPT, cognitive processing therapy; HR, hazard ratio; MH, mental health; NA, not applicable; PE, prolonged exposure.

^a^ Data on race and ethnicity were extracted from the US Veterans Affairs Corporate Data Warehouse. No further breakdown of the classifications is available.

## Discussion

This cohort study examined CPT/PE receipt and subsequent suicide mortality among a national cohort of veterans with newly diagnosed PTSD. Across analyses, we found that veterans who initiated CPT/PE had a lower risk of suicide compared with those who did not initiate CPT/PE. Although receipt of 8 or more CPT/PE encounters was associated with lower suicide risk in unadjusted analyses, this finding was not significant in adjusted analyses. However, the parameter estimates for adequate therapy (HR, 0.80) and initiation (HR, 0.77) were similar, and it is notable that CPT/PE initiators who received fewer than 8 sessions were included as controls in the analysis of adequate treatment analyses. Thus, differences in significance between the 2 analyses should be interpreted with caution. Study findings are consistent with clinical evidence supporting treatment-related reductions in psychopathologic diagnoses^16,17,18,19,20,21^ and suicidal ideation^23,24,25,26,27^ following initiation of CPT/PE. This study provides new information regarding associations with suicide mortality. These findings suggest the importance of initiation of CPT/PE as first-line treatments for PTSD; however, we did not find support for suicide risk reduction following an adequate course of therapy.

We also found that, among veterans who received some form of psychotherapy, those who initiated CPT/PE had lower suicide risks compared with those who only received other forms of psychotherapy. This is consistent with meta-analysis reports that CPT and PE are associated with greater decreases in PTSD symptoms than other forms of manualized therapy or treatment as usual.^13,14^ Although specific psychotherapies were not directly compared with CPT/PE in the present investigation, study findings suggest that reductions in suicide risk associated with CPT/PE receipt may be greater than the effects of psychotherapy generally among patients with new PTSD diagnoses who are veterans treated within the VHA.

We note that, although 65.2% of the cohort received psychotherapy, only 8.7% of the study cohort initiated CPT/PE, as documented by national CPT/PE note templates.^9^ Given that CPT/PE initiation was associated with a lower suicide risk among veterans who initiated any form of psychotherapy, it is important to discuss CPT and/or PE as part of shared treatment decision-making for all veterans with new PTSD diagnoses and understand barriers to initiating CPT/PE. These findings further highlight the need for continued efforts to ensure that veterans with PTSD have timely access to CPT/PE. National implementation of CPT and PE in the VA involved a multidimensional approach^53^ with specific strategies, such as providing health care professional training programs and appointing local evidence-based psychotherapy coordinators,^54^ identifying effective therapeutic program elements,^55^ implementing CPT/PE training programs,^56^ developing a PTSD treatment decision aid,^57^ and instituting policies ensuring that all veterans with PTSD have access to CPT or PE.^58^ These findings highlight the benefits of ensuring access to CPT and PE among veterans and the need for continued dissemination of these treatments, access to timely care, and educational programs to expand training on the use of CPT and PE for VHA health care professionals.

In addition to CPT/PE receipt, the number of mental health inpatient days and demographic indicators were associated with suicide risk. The presence of mental health inpatient stays has been identified as being associated with suicide in earlier work,^8,59^ and the time following discharge from inpatient stays has been identified as a particularly high-risk period.^60^ Additional demographic variables that were associated with suicide risk included younger age, male sex, White race, non-Hispanic ethnicity, non-Northeast region of residency, and nonmarried status. These demographic groups have been associated with suicide in earlier studies.^6,8^ However, the effect sizes in the present study should be interpreted with caution given the propensity adjustment for CPT/PE receipt. Together, these findings suggest the importance of considering variation in suicide risk among veterans with PTSD across strata of mental health inpatient use and demographic factors.

### Limitations

This study has several limitations. We were unable to differentiate between veterans in VHA care who were offered CPT/PE and declined vs those who were not offered these treatments. Although propensity adjustment was used to correct for the likelihood to receive CPT/PE, residual confounding may still be present. There may be unmeasured variables related to preferences for evidence-based PTSD treatments that could have influenced the associations between suicide and CPT/PE initiation and receipt of an adequate course of therapy. Replication studies are needed to confirm our findings. Additionally, analyses examining CPT/PE receipt among patients who received psychotherapy were potentially confounded by comorbid mental health conditions. For example, the presence of comorbid bipolar disorder may both increase a veteran’s suicide risk^6^ and decrease the likelihood that professionals initiate an evidence-based practice for PTSD in favor of evidence-based practices for bipolar disorder. Furthermore, we used nationally standardized CPT/PE note templates to identify CPT/PE sessions. Therefore, CPT/PE sessions that were not documented using the VHA’s standardized templates were not included. However, a previous study reported that 80% of CPT/PE sessions delivered in the VHA are documented using the standardized note templates,^61^ suggesting that most CPT/PE sessions were included in this study. Future work should include newly available note templates for eye movement desensitization and reprocessing therapy, an evidence-based treatment for PTSD that is currently being initiated in the VA. We defined adequate therapy as 8 or more sessions of CPT/PE; however, some patients may be early responders and complete therapy before receiving 8 sessions.^62^ We were unable to assess PTSD symptom change as related to suicide, given the sparse nature of symptom data in the health record.^63^ In addition, study data did not include detailed information about treatment adherence. Therefore, we were unable to assess whether treatment process factors that are associated with symptom reductions during CPT/PE (eg, homework completion^64,65^) were associated with reduced suicide risk among veterans.

## Conclusions

This cohort study provides national data regarding suicide risk among veterans in VHA care with new diagnoses of PTSD by receipt of evidence-based therapy and 8 or more CPT/PE sessions. Veterans who initiated CPT/PE had a lower suicide risk than those who did not, controlling for propensity to initiate CPT/PE and for other covariates. These findings were consistent across the cohort and for veterans who were receiving some form of psychotherapy. Study findings support recommendations of CPT/PE as treatment for veterans with new diagnoses of PTSD.

## Notes

Supplement 1. **eTable 1.** Descriptive Statistics for the Inverse Propensity Score Distribution in the Overall Cohort and by Receipt of CPT/PE **eTable 2.** Covariate Distribution Across CPT/PE and Non-CPT/PE Groups Before and After Inverse Probability Weighting **eFigure.** Distribution of Inverse Propensity Scores in the Overall Cohort and by Receipt of CPT/PE

Supplement 2.
Data Sharing Statement
