# Supplementary material for: Suicide Risk Among Veterans Who Receive Evidence-Based Therapy for Posttraumatic Stress Disorder
Source: JAMA Netw Open. 2024 Dec 26;7(12):e2452144. doi: 10.1001/jamanetworkopen.2024.52144 (PMC11672158; doi:10.1001/jamanetworkopen.2024.52144)
Supplement: Supplement 1. — eTable 1. Descriptive Statistics for the Inverse Propensity Score Distribution in the Overall Cohort and by Receipt of CPT/PE eTable 2. Covariate Distribution Across CPT/PE and Non-CPT/PE Groups Before and After Inverse Probability Weighting eFigure. Distribution of Inverse Propensity Scores in the Overall Cohort and by Receipt of CPT/PE [file jamanetwopen-e2452144-s001.pdf]

Supplementary Online Content

Saulnier KG, Brabbs S, Szymanski BR, Harpaz-Rotem I, McCarthy JF, Sripada RK. Suicide risk among veterans who receive evidence-based therapy for posttraumatic stress disorder. *JAMA Netw Open*. 2024;7(12):e2452144. doi:10.1001/jamanetworkopen.2024.52144

eTable 1. Descriptive Statistics for the Inverse Propensity Score Distribution in the Overall Cohort and by Receipt of CPT/PE

eTable 2. Covariate Distribution Across CPT/PE and Non-CPT/PE Groups Before and After Inverse Probability Weighting

eFigure. Distribution of Inverse Propensity Scores in the Overall Cohort and by Receipt of CPT/PE

This supplementary material has been provided by the authors to give readers additional information about their work.

© 2024 Saulnier KG et al. *JAMA Network Open.*

eTable 1. Descriptive statistics for the inverse propensity score distribution in the overall cohort and by receipt of CPT/PE.

Inverse Propensity Score Distribution	Mean	Standard Deviation	Minimum	25% Quartile	Median	75% Quartile	Maximum
Overall Cohort	19.13	16.85	1.48	8.82	14.07	23.52	578.94
Did Not Receive CPT/PE	19.88	17.26	1.53	9.25	14.72	24.51	578.94
Initiated CPT/PE	11.20	8.24	1.48	6.00	8.99	13.72	183.40

© 2024 Saulnier KG et al. *JAMA Network Open.*

eTable 2. Covariate distribution across CPT/PE and non-CPT/PE groups before and after inverse probability weighting.

	Unweighted by Inverse Propensity Score			Weighted by Inverse Propensity Score			
Variable	No CPT/PE	Initiated CPT/PE	Standardized Difference	No CPT/PE	Initiated CPT/PE	Standardized Difference	Absolute Standardized Change
Age	50.61	44.48	-0.41	58.27	48.74	-0.15	-0.25
Female (*vs. Male*)	0.13	0.22	0.24	0.06	0.14	0.27	0.03
PCL Proportion in Facility	0.54	0.54	0.01	0.54	0.54	0.00	<-0.01
Charlson Comorbidity Index	0.45	0.25	-0.22	0.82	0.40	-0.08	-0.14
PC-PTSD Screen Score	0.50	0.76	0.20	0.34	0.55	0.05	-0.15
Drive Time to Facility	19.96	18.81	-0.07	21.90	20.26	-0.02	-0.04
Comorbid Psychiatric Diagnosis	0.42	0.54	0.23	0.33	0.45	0.25	0.02
VA Medical Center (*vs. CBOC*)	0.50	0.58	0.16	0.44	0.51	0.14	-0.01
TBI Diagnosis	0.02	0.03	0.06	0.02	0.03	0.07	0.01
Non-White (*vs. White*)	0.28	0.31	0.06	0.25	0.29	0.10	0.04
Multiple Race (*vs. White*)	0.01	0.01	0.00	0.01	0.01	0.00	<0.01
Any ED Visit	0.20	0.20	0.00	0.22	0.22	-0.01	0.01
Any Psychiatric Inpatient Stay	0.02	0.03	0.05	0.02	0.03	0.08	0.02
Psychiatric Medication Receipt	0.53	0.50	-0.05	0.58	0.53	-0.10	0.05
Positive PHQ-9 Item 9	0.03	0.06	0.15	0.01	0.04	0.14	-0.01
Positive PHQ-2 Screen	0.08	0.12	0.12	0.06	0.09	0.11	-0.01
Service Connection > 30%	0.63	0.52	-0.23	0.74	0.62	-0.26	0.03
Married	0.51	0.50	0.00	0.53	0.51	0.02	0.02
Separated	0.05	0.05	-0.02	0.04	0.05	-0.02	<-0.01
Divorced	0.22	0.21	-0.02	0.23	0.23	-0.04	0.01
Never Married	0.18	0.20	0.07	0.15	0.19	0.12	0.06
Widowed	0.02	0.01	-0.09	0.04	0.02	-0.15	0.06
Urban Residence	0.71	0.74	0.07	0.66	0.72	0.13	0.06
Rural Residence	0.26	0.24	-0.06	0.30	0.25	-0.10	0.05
Highly Rural Residence	0.03	0.02	-0.05	0.04	0.03	-0.08	0.02
Insular Island Residence	0.00	0.00	0.00	0.00	0.00	0.00	<-0.01
Experienced MST	0.07	0.13	0.18	0.04	0.08	0.17	-0.01
Did Not Experience MST	0.78	0.68	-0.22	0.88	0.78	-0.27	0.05
Declined to Answer MST	0.00	0.00	0.01	0.00	0.00	0.01	<0.01

© 2024 Saulnier KG et al. *JAMA Network Open.*

Unknown Presence of MST	0.15	0.19	0.11	0.08	0.14	0.19	0.07
Served Pre-Gulf War Era	0.39	0.22	-0.38	0.61	0.35	-0.54	0.16
Served Post-Gulf War Era	0.61	0.78	0.38	0.39	0.65	0.54	0.16
Unknown Era of Service	0.01	0.00	-0.03	0.00	0.00	0.00	-0.03

Note. Absolute standardized change differences <0.10 indicate balance. PCL = PTSD Checklist for DSM. PC-PTSD Screen = Primary care posttraumatic stress disorder screen. VA = Veterans Affairs. CBOC = Community-based outpatient clinic. TBI = Traumatic brain injury. ED = Emergency department. PHQ-9 = Patient Health Questionnaire-9. PHQ-2 = Patient Health Questionnaire-2. MST = Military sexual trauma.

© 2024 Saulnier KG et al. *JAMA Network Open.*

eFigure. Distribution of inverse propensity scores in the overall cohort and by receipt of CPT/PE.

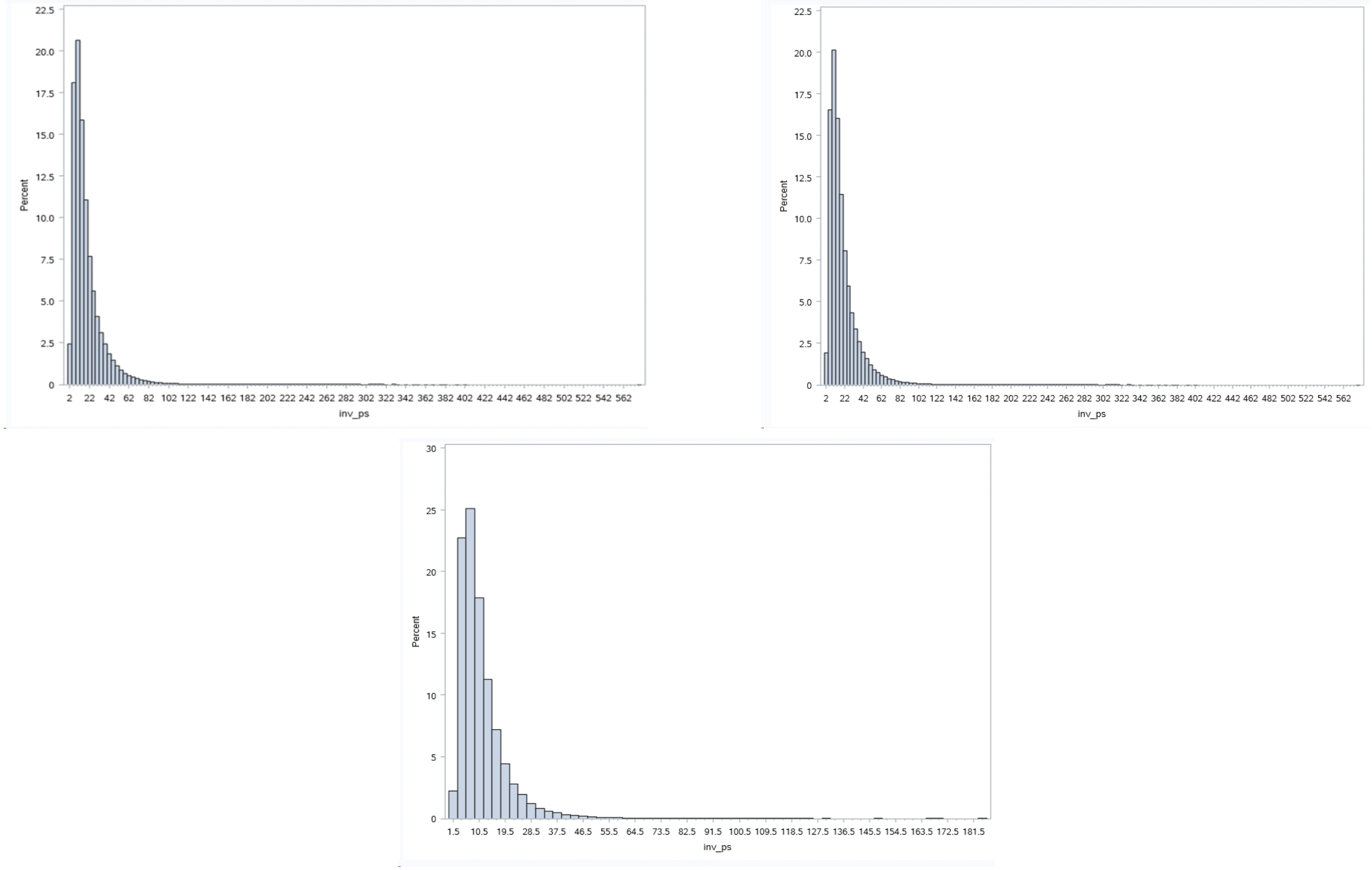

Note. Top left panel displays the distribution of inverse propensity scores in the overall cohort. Top right panel displays the distribution of inverse propensity scores among patients who did not receive CPT/PE. Bottom middle panel displays the distribution of inverse propensity scores among patients who received CPT/PE.

© 2024 Saulnier KG et al. *JAMA Network Open.*
