# Supplementary material for: Suicide Risk Among Veterans Who Receive Evidence-Based Therapy for Posttraumatic Stress Disorder
Source: JAMA Netw Open. 2024 Dec 26;7(12):e2452144. doi: 10.1001/jamanetworkopen.2024.52144 (PMC11672158; doi:10.1001/jamanetworkopen.2024.52144)
Supplement: Supplement 2. — Data Sharing Statement [file jamanetwopen-e2452144-s002.pdf]

Data Sharing Statement

Saulnier. Evidence-Based Therapy for Veterans With Posttraumatic Stress Disorder and Subsequent Suicide. *JAMA Netw Open*. Published December 26, 2024.
doi:10.1001/jamanetworkopen.2024.52144

Data
Data available: No
